# Editorial: Neuromechanical Biomarkers in Robot-Assisted Motor Rehabilitation

**DOI:** 10.3389/fnbot.2021.831113

**Published:** 2022-01-12

**Authors:** Andrés Úbeda, Alvaro Costa-Garcia, Diego Torricelli, Ivan Vujaklija, Alessandro Del Vecchio

**Affiliations:** ^1^Human Robotics Group, Department of Physics, Systems Engineering and Signal Theory, University of Alicante, Alicante, Spain; ^2^Intelligent Behaviour Control Unit, CBS-Toyota Collaboration Center, RIKEN, Nagoya, Japan; ^3^Instituto Cajal, Spanish National Research Council (CSIC), Madrid, Spain; ^4^Bionic and Rehabilitation Engineering Group, Department of Electrical Engineering and Automation, Aalto University, Espoo, Finland; ^5^Neuromuscular Physiology and Neural Interfacing Group, Department Artificial Intelligence in Biomedical Engineering, Friedrich-Alexander Universität, Erlangen-Nürnberg, Erlangen, Germany

**Keywords:** robot-assisted rehabilitation, neuromechanical biomarkers, electromyography, motor control, bioinspired devices

Clinical evaluation of motor function is essential for tracking the evolution of patient's abilities during rehabilitation. A regular and accurate observation of neuromotor recovery allows therapists to adjust intensity, number of repetitions, or targeted motor activity during the treatment, in particular when delivered using advanced technological means. However, conventional evaluation methods are usually based on qualitative clinical metrics with low resolution (Hsieh et al., 2009) and, commonly, the correct interpretation of the relevant scales depends on the experience of the attending therapist. Consequently, the assessment of functional recovery suffers the risk of being incomplete or inaccurate. This editorial aimed to prompt and gather a collection of novel research efforts with the common goal of identifying relevant or promising neuromechanical biomarkers of neuromotor functions during robot-assisted rehabilitation in clinical settings.

Indeed, the latest rehabilitation technologies allow, through the combination of sensors and robots, to measure the patient's kinematic and/or kinetic movement parameters with high precision (Bosecker et al., 2010; Colombo et al., 2015; Keller et al., 2015). These parameters can be used to assess motor learning or quantify improvements in targeted motor functions. Furthermore, the same technologies allow to investigate how the neuromuscular system is behaving from an electrophysiological point of view. As reviewed in detail by Garro et al. current research is mostly based on surface electromyography (EMG) and electroencephalography (EEG). Some studies already correlate these metrics with conventional clinical scales (Tang et al., 2018; Zhang et al., 2019), but their application to the optimization of robot-assisted rehabilitation has not yet been systematically explored.

In this context, several examples of clinical estimation of neuromechanical biomarkers are explored in this collection. The aforementioned review by Garro et al. analyzes a number of non-invasive electrophysiological approaches to the computation of biomarkers from EEG and EMG recordings, particularly focused on stroke and robot-assisted rehabilitation. In Ye et al. data-driven models based on backpropagation neural networks (BPNN) are built from EMG data collected from chronic stroke individuals and correlated with the Fugl-Meyer Assessment scale (FMA) and the Modified Ashworth Scale (MAS). Ezaki et al. evaluate joint angles and muscle activity during gait before and after intervention with a HAL exoskeleton, reporting changes in acute and chronic patients with Ossification of the Posterior Longitudinal Ligament (OPLL) caused by myelopathy. In Reyes et al. variations in the Metabolic Equivalent of Task (METs) are found under different conditions of friction during walking activities using a Motor Assisted Hybrid Neuroprosthesis (MAHNP). Finally, in Longatelli et al. functional gait is assessed in terms of neuromuscular behavior during exoskeleton training, showing that patients treated with the robotic device regained a controlled rhythmic neuromuscular pattern in the proximal lower limb muscles.

The presented articles in this editorial give insights into the estimation of neuromechanical biomarkers in clinical scenarios in a non-invasive way and with robustness similar or higher to that of conventional clinical scales. These studies illustrate some of the future directions in this field arguably indicating the trend of neuromechanical assessment in clinical motor rehabilitation.

## Author Contributions

AÚ drafted the first version of the manuscript. All authors contributed to the critical discussion and revision of its contents.

## Conflict of Interest

The authors declare that the research was conducted in the absence of any commercial or financial relationships that could be construed as a potential conflict of interest.

## Publisher's Note

All claims expressed in this article are solely those of the authors and do not necessarily represent those of their affiliated organizations, or those of the publisher, the editors and the reviewers. Any product that may be evaluated in this article, or claim that may be made by its manufacturer, is not guaranteed or endorsed by the publisher.
